# Open Tubular Liquid Chromatography and the Analysis of Single Neurons

**DOI:** 10.6028/jres.093.094

**Published:** 1988-06-01

**Authors:** J. W. Jorgenson, R. T. Kennedy, R. L. St. Claire, J. G. White, P. R. Dluzneski, J. S. M. de Wit

**Affiliations:** Department of Chemistry, The University of North Carolina, Chapel Hill, NC 27514

Liquid chromatography in open tubular columns (OTLC) offers a means of achieving separations of high resolving power within analysis times of minutes to hours. A theory which predicts the optimal dimensions for an open tubular column for a given set of analytical conditions has been developed [1]. This theory predicts that for a wide range of possible inlet pressures and analysis times the most efficient columns will result when the column inner diameter is between 1.5 and 3 µm. A column of 2 µm diameter and 2 meter length should be capable of producing a million theoretical plates for an analyte with a capacity factor of 10 (strongly retained) and a retention time of 100 minutes.

## Mobile Phase Delivery and Sample Injections

### Mobile Phase Delivery

For columns of a few micrometers internal diameter, the flow rate of mobile phase is on the order of picoliters per second. Such flow rates are far below the capabilities of any existing commercial pumps. A simple system which uses the constant head pressure of helium gas for pumping mobile phase in OTLC columns has been described [1]. This system makes use of the fact that the flow impedance of the open tubular column is constant under isocratic conditions, and thus a constant head pressure yields a constant flow rate. For gradient elution purposes, a commercial gradient pumping system used with a solvent splitting arrangement has been described by Yang [2]. This system permits gradient elution for micro-packed columns and is applicable to open tubular columns as well. A minor amount of solvent waste is the only disadvantage of such a solvent splitting system.

### Sample Injections

A number of schemes for introducing samples into microcolumns have been described. As the inner diameter of the column is reduced, the need for low dispersion in the introduction process becomes more critical. A simple “static splitting” system which directs a small portion of sample to the column has been described [1]. This system is assembled from standard commercial chromatographic plumbing and is capable of injecting sample plugs of essentially any desired length onto columns of any internal diameter (the chromatogram shown in figure 2 was obtained from a 7 picoliter injection onto a 1.7 µm diameter column using this system). The length of the injected plug is controlled by the flow rate of mobile phase through the column and the timing of injection valves. The main disadvantage of this system is that sample volumes of approximately half a milliliter are required to fill the injection “loop” even though only a few picoliters may actually be injected. Judicious engineering of the system could reduce the volume of sample required to perhaps 10 microliters. An alternative approach capable of injecting subnanoliter volumes of sample will be described in the section on single cell analysis.

## Column Preparation

A major problem in OTLC systems is the preparation of columns with sufficient stationary phase. Capillary tubing in micrometer sized dimensions is readily available, but successfully “coating” large enough amounts of stationary phase into the capillary is a serious challenge. We have prepared columns of two basic types. The first is based on borosilicate glass capillaries in which a superficially porous layer is formed on the surface by chemical leaching. Silane stationary phases may be subsequently bonded to this porous layer. As an alternative, columns may be prepared by depositing polymeric stationary phase on the inner wall of fused silica capillaries.

### Porous Layer Glass Capillaries

Capillaries are drawn from borosilicate glass tubing using a conventional glass drawing machine. They are then heat treated at 600 °C for 24 hours, inducing a phase separation in the glass. Following heat treatment, the column is leached with a hydrochloric acid solution. This renders the surface porous by selectively removing the non-silica components of the glass and leaving behind a porous silica layer. This process increases the internal surface area of the capillary roughly thirtyfold over a geometrically smooth capillary. This surface is then suitable for bonding of silane stationary phases [1,3,4].

### Polymer Layer Fused Silica Capillaries

Fused silica capillaries with internal diameters as small as 5 µm are now commercially available. Capillaries of fused silica offer the advantages of greater flexibility and resistance to breakage over conventional glass capillaries. They also exhibit better UV transparency and lower intrinsic luminescence than borosilicate glass, significant advantages when on-column fluorescence detection is to be done. Unfortunately, little is known about methods to increase the internal surface area of fused silica in order to bond sufficient amounts of silane stationary phase. As an alternate approach we have devised a method for coating siloxane polymer stationary phases onto the surface of these capillaries [5,6]. The method relies on the fact that the polymer solubility is temperature dependent. In this procedure OV-17v, a polysiloxane with 49% phenyl, 49% methyl, and 2% vinyl substitution, is dissolved along with a small amount of benzoyl peroxide (as a cross linking reagent) into a solvent composed of 70% heptane and 30% cyclohexane at a temperature of 80 °C. The polymer and peroxide are both quite soluble in this solvent system at this temperature. The capillary is then filled with this solution while maintaining the capillary at the elevated temperature. After filling, the capillary is simply allowed to cool to room temperature, whereupon the polymer and peroxide “coprecipitate” onto the capillary surface. The remaining solvent is flushed from the capillary with helium gas and the column is slowly heated to 140 °C at which point the benzoyl peroxide decomposes to generate free radicals which initiate cross linking of the polymer chains. This produces an immobile and inextractable polymer layer which is useful as a stationary phase.

## Detection

The tiny dimensions and high efficiency of OTLC columns dictate that detectors be extremely sensitive and contribute a minimum of additional dispersion to the chromatographic peaks. We have developed detectors for on-column fluorescence detection as well as on-column electrochemical detection. Post column gas phase photoionization detection [7] and mass spectrometric detection [8] have been developed as well.

### Fluorescence Detection

**Figure 1:**
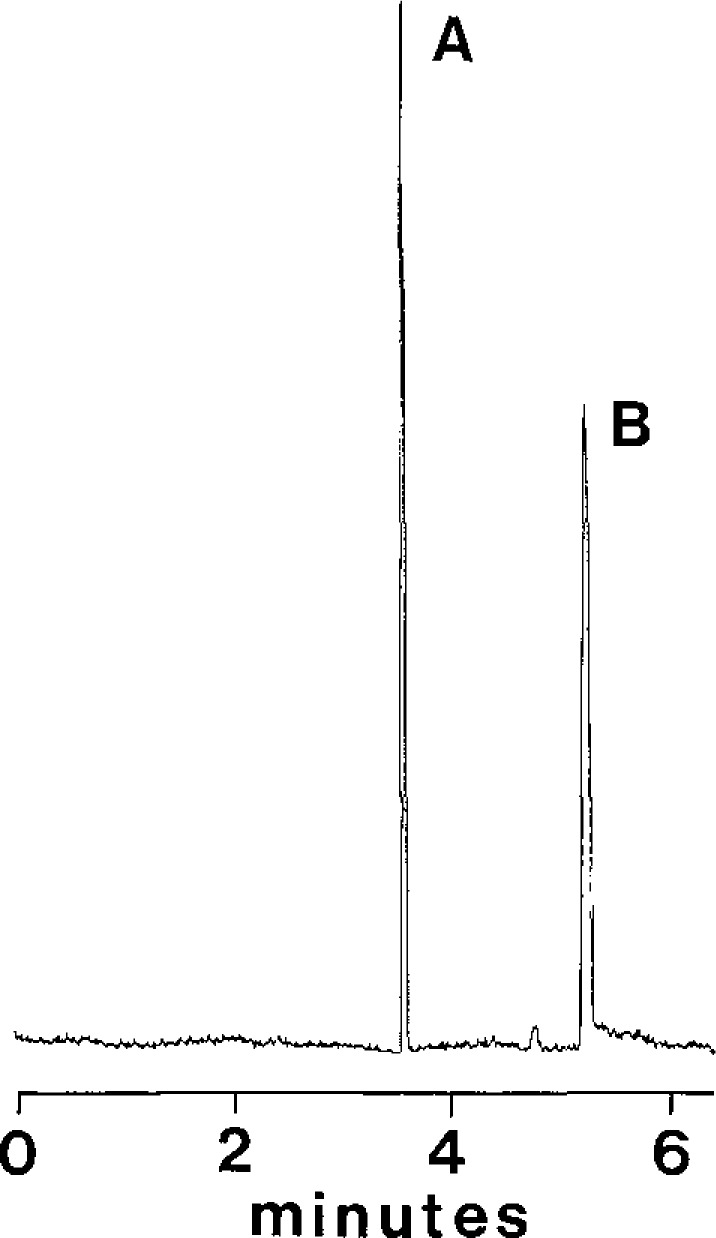
Chromatogram of riboflavin (A) and perylene (B) run on a 1.7 µm diameter fused silica column.

On-column fluorescence detection with a conventional arc lamp source [9] offers acceptable sensitivity with columns of diameters larger than 10 µm, but smaller capillaries require the use of lasers as sources [10]. Figure 1 is a chromatogram obtained on a 1.7 µm diameter fused silica column with a 7 picoliter injection of a solution 1 micromolar in both riboflavin and perylene. This corresponds to only 7 attomoles or 4 million molecules injected onto the capillary. The detection limit in this case is only 100,000 molecules, attesting to the high sensitivity possible with laser induced fluorescence detection.

### Electrochemical Detection

**Figure 2:**
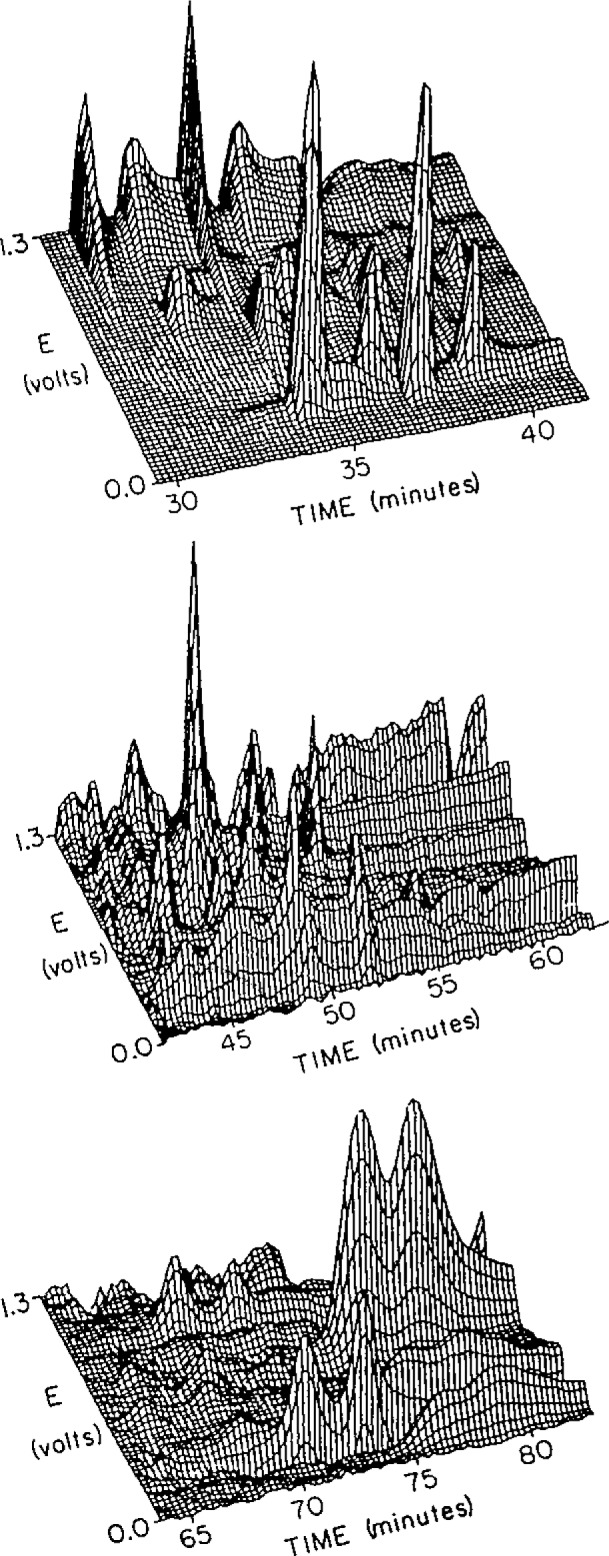
Chromatovoltammogram of a sample of tea, run on a 15 µm borosilicate reversed phase column.

An on-column electrochemical detector based on a single carbon fiber as the working electrode has been developed. The fiber electrode is inserted into the outlet of the OTLC column with the aid of a micropositioner and a microscope. Current may be measured at a fixed potential (amperometric) [11,12] or the voltage may be scanned and current measured as a function of applied potential (voltammetric) with the aid of a microcomputer [13,14]. The minimum detectable quantity of analyte is roughly 10 attomoles in the amperometric mode and 100 attomoles in the voltammetric mode. Figure 2 is a chromatovoltammogram of a sample of tea obtained on a 15 µm reversed phase borosilicate glass capillary [15]. The additional dimension of “separation” offered by voltammetry may be a significant advantage when working with such a complex sample.

## Analysis of Single Neurons

**Figure 3:**
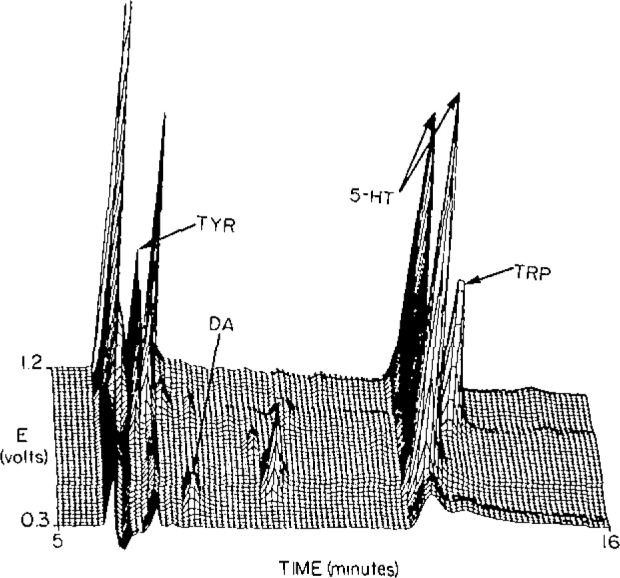
Chromatovoltammogram of the contents of a single neuron. Compounds identified are tyrosine (TYR), dopamine (DA), serotonin (5-HT), and tryptophan (TRP).

The small sample requirements and high sensitivity of the OTLC systems just described make them ideal for analysis of samples of small volume, such as the contents of individual cells. We have developed a method which permits us to isolate individual giant (120 µm) neurons from various ganglia of the land snail, *Helix aspersa*. These neurons are used because of their large size and because they can be reproducibly located from specimen to specimen. A single neuron is transferred to a 500 nanoliter microvial. It is disrupted and homogenized with a glass fiber, its contents centrifuged, and the supernatant drawn up into a micropipette. The tip of the micropipette is then inserted into the inlet end of an OTLC column and the cellular fluid [16]. Figure 3 is a chromatovoltammogram obtained from the neuron designated F76 (anatomical map of Kerkut et al. [17]). Four compounds, tyrosine, dopamine, serotonin, and tryptophan, have been identified and quantified in several different giant neurons of the snail. Finding two classical neurotransmitters, dopamine and serotonin, in the same cell is an interesting result. This analysis is made feasible by the ability of OTLC to work with exceptionally small samples.
